# Reply to Sullins (2023) on the Ethical and Human Rights Concerns of Sexual Orientation Change Efforts

**DOI:** 10.1007/s10508-023-02620-8

**Published:** 2023-05-30

**Authors:** Jenna Marie Strizzi, Ezio Di Nucci

**Affiliations:** Department of Public Health, University of Copenhagen, Øster Farimagsgade 5, 1014 København K, Copenhagen, Denmark

We must respond to Sullins’ (2023) reply to Strizzi and Di Nucci (2023), for two reasons: Sullins brings up some plausible arguments that we would like to engage and further elaborate on, but Sullins also accuses us of censorship, which we must resist in the strongest terms.

First, we would like to anchor the focus on the topic at hand, Sexual Orientation Change Efforts (SOCE). SOCE includes a wide range of techniques including:

talk therapy or psychotherapy (e.g., exploring life events to identify the cause); group therapy; medication (including anti-psychotics, anti-depressants, anti-anxiety, psychoactive drugs, and hormone injections); Eye Movement Desensitization and Reprocessing (where an individual focuses on a traumatic memory while simultaneously experiencing bilateral stimulation); electroshock or electroconvulsive therapy (ECT) (where electrodes are attached to the head and electric current is passed between them to induce seizure); *aversive treatments (including electric shock to the hands and/or genitals or nausea-inducing medication administered with presentation of homoerotic stimuli);* exorcism or ritual cleansing (e.g., *beating the individual with a broomstick while reading holy verses or burning the individual’s head, back, and palms*); *force-feeding or food deprivation; forced nudity; behavioural conditioning (e.g., being forced to dress or walk in a particular way); isolation (sometimes for long periods of time, which may include solitary confinement or being kept from interacting with the outside world); verbal abuse; humiliation; hypnosis; hospital confinement; beatings; and “corrective” rape* (Alempijevic et al., 2020, p. 66–67, italics added for emphasis).

As SOCE can and does include extremely violent techniques, SOCE has been likened to some forms of torture (Alempijevic et al., 2020; Nugraha, 2017). Applying the principle of charity, we are happy to give the other side the benefit of the doubt, but an inclusive understanding of SOCE is obviously unethical, independent of outcomes, and Sullins has failed to offer a stricter definition, with, for example, precise conditions about informed consent and adaptive preferences. We sincerely hope for Sullins’ “moral health” that when he argues for overturning the “false narrative” that SOCE is harmful and that restrictions should be reconsidered (because, according to his analyses, SOCE reduce suicidality [Sullins, 2022, 2023]) that he is not advocating for the raping and/or beating of individuals in an attempt to make them straight/heterosexual. However, the potential for interpreting his work (Sullins, 2022) to justify these practices and violence against sexual minority populations is real.

Moreover, the data from the Generations Study do not specify the types of SOCE techniques that participants were exposed to (Blosnich et al., 2020; Sullins, 2022). The Generations Study was conducted in the context of the USA and violence is not reported to be a common SOCE practice in that country (Alempijevic et al., 2020) and the most common SOCE practice in that country is psychotherapy (Shidlo & Schroeder, 2002). Given that it is impossible to ascertain whether participants had been exposed to violence with the data employed, it makes Sullins’ (2022, 2023) claims that SOCE reduces suicidality and that restrictions against these practices should be reconsidered particularly problematic as it can be easily interpreted (and used) to support all or any SOCE practice conducted in any context, in the USA and/or globally, including violence against (and the torturing of) sexual minority individuals.

Further, it seems there are some issues with reading comprehension (or willful misinterpretation?) of our original commentary. Sullins asserts that we “call for censorship of positive or even neutral SOCE findings” which is categorically untrue. We stated that:

Our main argument here is that the obvious and very serious ethical and human rights concerns related to SOCE transcend any methodological analysis by Sullins (2022) or indeed anyone else, for the simple reason that the problem with SOCE is not just about outcomes and well-being but primarily about rights and autonomy so that a methodological analysis seeking to undermine causation is just irrelevant (Strizzi & Di Nucci, 2023, p. 865).

We look forward to Sullins engaging with our main argument above but are still waiting. The key point in our original commentary—and what we reiterate here—is that when discussing an issue such as SOCE, regardless of one’s religious or political inclinations, the problem with SOCE is fundamentally about ethics and sexual and human rights. Sullins (2022, 2023) claims that SOCE reduces suicidality and thus restrictions against SOCE ought to be revoked or reconsidered to protect the well-being of sexual minority populations. There are several issues with this line of reasoning. What we mean when we state that any analysis of the possible link between SOCE and suicidality is secondary and irrelevant (considering that the human rights concerns outweigh and transcend the direction or strength of the link) is that, regardless of the possible link between the behavior (SOCE) and the outcome (suicidality), the severity of human rights violations of the behavior (SOCE) cannot justify the outcome (in this case, reduced suicidality).

Let us spell this out a bit more: SOCE is likened to torture as it can (and often does) constitute torture (Alempijevic et al., 2020; Nugraha, 2017). Now consider Sullins’ (2022, 2023) conclusions employing this language (SOCE → torture). Following Sullins’ (2022, 2023) flawed reasoning, if analyses of data revealed that the behavior (torture) reduces suicidality, then restrictions on torture should be lifted and torture may, in fact, be beneficial to the well-being of those who are tortured. A reasonable person will find that this logic is outrageous and agree that the human rights concerns of the behavior or practice outweigh whether the association between the variables is statistically significant or, in other words, that might be familiar beyond philosophical circles, the end does not always justify the means. This is what we argued originally and we stand by that and look forward to Sullins’ counterargument, in the traditional fashion of scientific debate.

Sullins’ (2023) concerns about censorship are important and we agree that limiting free speech and censorship can be problematic in general and in science. We do not call for a limitation of his or anyone else’s rights to free speech or academic freedom. We simply state that, in our view, the human and sexual rights violations associated with SOCE should be considered central in the study of, and debate and discussion about, this topic. For example, if we replace the language describing the behavior (SOCE → torture) and maintain the outcome (suicidality), understanding the relationships between the behavior and the outcome from a scientific view is of value, public health, and public policy relevance. In this same example, torture, as we did with SOCE, we do not call for a limitation of science to understand the public health implications of these behaviors or practices. What we intended to communicate with “any work advocating human rights violations, which also constitute unethical and fraudulent treatment of individuals or patients, has no place in scientific discourse” (Strizzi & Di Nucci, 2023, p. 866), which we assume may have been interpreted as a call for censorship, was not to censor scientific inquiry in the area of SOCE but to precisely caution against advocating for human rights violations.

It could be that Sulllins is referring to non-violent SOCE techniques (which also pose ethical and human rights concerns), but as SOCE includes a wide range of practices, his claims can be easily interpreted as (and problematically used for the purposes of) advocating for violence against sexual minority individuals (i.e., lifting bans and/or restrictions on SOCE, including the practices described above like “corrective” rape). We hope that, regardless of one’s religious or political ideologies, we can all agree that this indeed has no place in scientific discourse. We agree with Sullins’ concerns about censorship, free speech, academic freedom, and religious freedom and do not advocate for censorship nor limitation of free speech other than the most widely accepted understandings of these concepts. Violence and calls for violence are not protected under freedom of speech and freedom of religion, nor should they be.

We appreciate Sullins’ discussion of self-determination and ardently support individuals’ right to absolute sexual self-determination. What is more, we ardently support an individual’s right to choose their sexual orientation and the right to change one’s sexual orientation. However, self-determination regards the freedom to make choices free of compulsion. Given the pervasiveness and high levels of heterosexism and anti-LGB stigma, it is difficult to categorically claim that there is no compulsion or coercion in SOCE.

Now, we come back to the central topic at hand, SOCE. As discussed above, the human rights concerns with these practices transcend the debate. Further, as Sullins’ (2022) clearly admits, SOCE are known to be ineffective at changing sexual orientation, especially long term, or at least there is not sufficient empirical support for long-term effectiveness. Thus, the discussion of self-determination and changing/choosing one’s sexual orientation by means of SOCE is simply not possible. As the ineffectiveness brings us to the topic of ethical treatment of patients and fraud, as these efforts are not effective, they constitute unethical treatment of patients and this compounds the concerns about SOCE.

We also appreciate Sullins’ (2023) discussion of religious freedoms as it brings to the surface a wider debate regarding sexual and human rights, cultural relativism, and religious freedom. Within philosophy and beyond, this is the classic debate of universal human rights vs. cultural relativism. It is polemic and controversial when religious and cultural beliefs confront sexual and human rights. Examples include male circumcision, female genital cutting, and forced virginity tests. These are larger philosophical questions: Do religious beliefs or cultural relativism justify human or sexual rights violations? Do cultural practices and religious beliefs transcend human and sexual rights? This is likely not the forum for this discussion and these questions cannot be resolved here.

Regarding the issue of pathology and sexual orientation, Sullins (2023) states that “It is not so simple a matter as declaiming, as if it were a universal truth, that same-sex attractions ‘are not considered pathologies’ (Strizzi & Di Nucci, 2023). It depends who is doing the considering. Religious teachings subscribed to by over half the planet consider same-sex relations morally unhealthful in various degrees” (p. 897). From a scientific perspective, we are comfortable in claiming that it is as simple as affirming as a universal truth that same-sex attractions and behaviors are not pathologies as the scientific community and decades of scientific evidence support this assertion.

Sullins (2023) concludes by urging for “respecting each other’s differences” (p. 898) but we inquire: Is it impossible to defend the claim of respecting differences while supporting and advocating for systematic efforts to homogenize the population by eradicating sexual minorities through sexual orientation change efforts?
